# Dopamine Receptor Gene *DRD4* 7-Repeat Allele X Maternal Sensitivity Interaction on Child Externalizing Behavior Problems: Independent Replication of Effects at 18 Months

**DOI:** 10.1371/journal.pone.0160473

**Published:** 2016-08-05

**Authors:** Anthony P. King, Maria Muzik, Lindsay Hamilton, Alexander B. Taylor, Katherine L. Rosenblum, Israel Liberzon

**Affiliations:** 1 Psychiatry, University of Michigan, Ann Arbor, Michigan, United States of America; 2 VA Ann Arbor Health System, Ann Arbor, Michigan, United States of America; 3 Center for Human Growth & Development, Ann Arbor, Michigan, United States of America; 4 Psychology, Bowling Green State University, Bowling Green, Ohio, United States of America; 5 Ecology and Evolutionary Biology, University of Michigan, Ann Arbor, Michigan, United States of America; University of Rennes-1, FRANCE

## Abstract

The *DRD4* VNTR has been associated with child behavior problems in interaction with maternal insensitivity in European and American cohorts of preschoolers, with the 7-repeat (7R) allele associated with greater problems. We sought to replicate and expand these findings by examining effects on reports of child behavior problems at 18 months. A 63 family sample with data for observed maternal sensitivity ratings, *DRD4* VNTR genotype, and maternal report of child behavior problems at 18-months was used in this preliminary analysis. Maternal sensitivity was measured at 6-months of age using laboratory observational measures (free-play and a teaching task). Maternal report of toddler behavior was obtained at 18-months via the standard Child Behavior Checklist, and infant genotype on the *DRD4* VNTR was obtained using PCR. Infants carrying the *DRD4* 7R allele showed greater effects of maternal insensitivity than non-carriers for behavioral problems at 18-months. We replicated previous findings of association of infant *DRD4* x maternal sensitivity interactions with child Externalizing problems in the European-ancestry sample (N = 42) in a median split of maternal sensitivity (p = .00011, eta2 = .329) and in regression analyses controlling for maternal age, maternal depression, and child gender in European ancestry (B = -3.4, SE 1.33, p = .01) and the total sample (B = -2.2, SE 1.02, p = .02). Exploratory analyses also found evidence of DRD4 x maternal sensitivity interaction with the CBCL ADHD scale. These findings replicate in an independent cohort *DRD4* x maternal insensitivity interaction effect on child externalizing behavior problems at 18 months, further supporting the role of the *DRD4* genotype in differential sensitivity to parenting.

## Introduction

Early life measures of behavioral problems have been associated with increased risk for problematic adjustment across a range of developmental domains (see review [1]). Externalizing behaviors observed in early childhood, in particular, have been associated with increased incidence of a range of problem outcomes, including increased risk for difficulties with peer relationships, academic competence, aggression, and criminality (e.g., [2,3]. Early parenting behavior, including parenting sensitivity, or the capacity to respond to appropriately to the child’s cues and needs, appears to play an important role in shaping child developmental pathways (Belsky & Jaffe, 2006). For example, higher levels of maternal sensitivity have been associated with better emotional regulation and lower levels of externalizing behaviors in infants and preschoolers [4,5]

Twin studies indicate genetics play important roles in early behavioral and emotional development, but the nature of genetic influences need elucidation. The dopamine system is strongly implicated in reward processing and approach behaviors [6]. A variable number of tandem repeats (VNTR) polymorphism in the dopamine D4 receptor gene (*DRD4*) is associated with structural and functional changes in the *DRD4* receptor. The 7-repeat allele (7R) codes for a receptor with lower biochemical activity and has been associated with various behaviors including addiction, gambling, and approach traits in a large number of adult studies, but meta-analyses that have not reliably validated all of the findings [7,8]. A meta-analysis did find evidence for association of the *DRD4* 7R allele with ADHD diagnosis in persons of European ancestry [9] and gene association studies in children have also reported association of the *DRD4* gene with aggression [10–13].

Gene x early childhood environment interaction is a potentially important mechanism by which child behavior is shaped [14]. Since the seminal studies of [15] on the serotonin receptor gene 5-HTTLPR variant, a number of studies have reported interactions with child maltreatment with 5-HTTLPR and other genes such as *FKBP5* and *ADRB2*, increasing vulnerability adult depression and PTSD [16–18] Gene x environment interactions have also been reported in children with less severe forms of environmental influences, such as the style of parenting behaviors. For example, the *DRD4* 7-repeat allele appears to moderate the effects of insensitive parental caretaking interactions on problem behaviors in kindergarten [19], greater inattention in middle childhood [20] and greater ADHD, conduct disorder, and psychopathy symptoms at age 15 [21].

A small number of studies have shown evidence that the *DRD4* 7-repeat allele moderates the effects of low levels of maternal sensitivity specifically on early childhood externalizing behaviors. Bakermans-Kranenburg & van Ijzendoorn [22] found a *DRD4* 7-repeat x maternal sensitivity (measured at 10 months of age) interaction that predicted externalizing behaviors at 39 months in an initial cohort of Netherlands toddlers. This group then replicated these findings in a larger longitudinal cohort, measuring maternal sensitivity at 14, 36, and 48-months, and externalizing behaviors at 18, 36, and 48 month old children [23]. Similar findings were found in European-ancestry, but not African-ancestry children [24]. Interestingly, a positive parenting intervention was also reported to be more effective in children carrying the *DRD4* 7R allele [25]

This paper was designed as an attempt to independently replicate *DRD4* 7-repeat allele x maternal interactive sensitivity on early childhood externalizing behaviors, using comparable observational measures of maternal sensitivity and similar analytic strategy, but focusing on observed maternal behaviors at an earlier time-point than previous studies (i.e., at 6 months of child age) and predicting to child externalizing behaviors at 18 months. Thus this study adds to the current literature a focus on very early risk behaviors in mother and child, both of which are known to be foundational for problematic developmental pathways [1].

## Methods

### Participants

Participants were drawn from the Maternal Anxiety during the Childbearing Years (“MACY” K23 MH080147), a longitudinal study of the effects of maternal psychopathology and childhood trauma history on infants’ biological and socio-emotional development. Eligible mothers were >18 years with babies < 6 weeks premature and no medical illnesses or developmental disabilities. A subsample of 63 mother-infant dyads with complete data on maternal sensitivity coding, child *DRD4* genotype, and 18 month CBCL was used for these analyses. The subsample was selected solely upon the availability of genetic specimen for genotyping. Collection of genetic specimens was added to the study after recruitment had started; thus inclusion (or lack of inclusion) in the subsample did not appear to reflect preferential retention or other selective pressures on inclusion. The subsample for which genetic specimens were available (N = 63) did not significantly differ from the rest of the sample (N = 205) on maternal age, maternal depression, maternal interactive sensitivity, or child CBCL measures.

### Data Access

All data used in the following analyses are publically available through the University of Michigan data archive “Deep Blue”, http://www.deepblue.lib.umich.edu/)

### Procedures

All procedures were approved by the University of Michigan Medical School IRB. Mother’s report of her own current depression symptoms, videotaped interaction tasks between mother and infant for assessment of maternal sensitivity, and saliva for genomic DNA were collected at a 6 months post-partum home visit. Maternal report of child behavioral problems were obtained at 18 months post-partum.

**Maternal Depression** was assessed via the previously validated 35-item Postpartum Depression Screening Scale (PPDS; [26]). Scores range from 35–175 with higher scores indicating more severe depressive symptoms (cut off for probable depression diagnosis is 80).

**Maternal Interactive Sensitivity** was evaluated at the 6-month home visit from videotapes of mother—infant interaction during two successive tasks varying in level of challenge: a 5-minute free play session, in which mothers were instructed to play with their child “as they normally would at home”, and two 3-minute “teaching tasks”, in which mothers were instructed to teach their infants developmentally difficult tasks (putting balls in a bucket and stacking blocks). Multiple dimensions of maternal behavior and affect were coded across interactive contexts using 5-point Likert scales from the MACY Infant—Parent Coding System (MIPCS; [27,28], including two components of maternal parenting sensitivity.

In our coding system (MACY Infant-Parent Coding System (Earls, Muzik, Beeghly, 2009), we captured maternal sensitivity twofold, both as behavioral and affective components. *Behavioral Sensitivity* taps into the mother’s awareness of and ability to perceive and respond to infant cues, manifested in well-timed vocalizations, facial expressions and physical handling responses that reflect empathy with the child’s needs and feelings. Mothers high in behavioral sensitivity almost always respond to their child’s cues in a timely and supportive matter, those low in behavioral sensitivity make few attempts to follow the child’s lead, show low awareness of her child’s cues, and/or may be intrusive or withdrawn. A score of 2 indicates some sensitivity, such that this mother, in general, sometimes responds to her infants signals, although she misses the more subtle ones, or responds after a moderate delay, whereas a score of 3 (or 4) captures a mother, who, in general, responds about half the time (or more than half the time, respectively) to infant’s signals, although she misses the other half of the signals, or responds after a short delay. *Affective Sensitivity* reflects the mother’s attunement with and empathy for her child’s subjective experience, as evidenced by the mother’s comments about and sharing of the child’s experience. Mothers high in affective sensitivity consistently exhibit understanding or empathy for her child’s internal experience, those low in affective sensitivity do not reflect or mirror their child’s affective experience. A score of 2 indicates some affective sensitivity, such that this mother, in general, mostly does not understand her infant’s affect, intentions, motives, or wishes, but may elicit a few instances of understanding or empathy; whereas, a score of 3 (or 4) indicates that she understands her infant’s affect, intentions, motives, or wishes half of the time (or more than half of the time, respectively), and demonstrates instances of understanding or empathy half of the time (or more than half of the time).

Given high correlation between behavioral and affective sensitivity scales (r(63) = 0.991, p < .001), we computed a summary variable, Maternal Parenting Sensitivity by averaging across both rating scales across all tasks, Scoring was conducted by trained coders who were masked to study variables. Inter-coder reliability with the primary coder was assessed using intra-class correlations (ICC) on 40 randomly selected videotapes independently scored by a trained, blinded coder. Maternal behavior scales had good to excellent inter-coder reliability (ICC across all tasks .85 to 0.86).

**Child Behavior Problems** were assessed by maternal report when infants were 18-months-old using the Achenbach CBCL for ages 1.5 to 5 [29]. The 113 items CBCL contains subscales for Externalizing Behaviors (Aggressive and Hyperactive), and Internalizing Behavior (Emotionally Reactive, Anxious/Depressed, Somatic Complaints, and Withdrawn), and Sleep problems, as well as DSM oriented scales of Attention Deficit/ Hyperactivity (ADHD), Oppositional Defiant, Affective, Anxiety, and Pervasive Developmental Problems.

**Infant DRD4 Genotype** Genomic DNA specimens were obtained from saliva using Oragene and purified using a semi-automated column method (QuickGene, Autogen Inc.) and quantified by OD_260_. The *DRD4* exon 3 fragment polymorphic VNTR region was amplified with PCR, as previously described [22], and alleles (number of repeats) for each sample were resolved by size fractionating of the PCR products with 2% agarose gel electrophoresis. The main *DRD4* genotypes in the sample (2/4, 4/4, 4/7) were in Hardy-Weinberg equilibrium. Children were grouped in subgroups of 7-allele “carriers”, i.e. carrying at least one 7-repeat allele, or “non-carriers”, those who did not have a 7-repeat allele.

### Statistical Analyses

Statistical analyses were performed using IBM-SPSS version 22.0. The primary analysis (replication of DRD4 x maternal sensitivity effect on child externalizing behaviors) followed the format of the original report [22], and used a median split of observed maternal sensitivity score in ANOVA with *DRD4* genotype in the portion of the sample with European-only ancestry (N = 42) and then in the combined sample (N = 63). Secondary analyses examined a *DRD4* x maternal sensitivity multiplicative interaction term in linear regression models also adjusting for maternal age, maternal depression at 18 months, child gender, and child ancestry (in the total sample), using bootstrapping (1000 bootstrap samples. Regions of significance (RoS) of the *DRD4* x maternal sensitivity interaction were calculated as previously described using a web-based tool (http://www.yourpersonality.net/interaction/) [30]. This provides a test for the specific values of the independent variable (maternal sensitivity) below and above which the regression lines for DRD4 7-allele carriers and non-carriers differ significantly in terms of child externalizing behaviors. This approach has previously been utilized [30–32] to examine whether the form of observed interactions are more consistent with a differential susceptibility model (disordinal [or “cross-over”] interactions in which significant differences are found on both positive and negative ranges [i.e. mean ± two SD] of the independent variable), or a diathesis-stress model (ordinal [or “spreading”] interactions, in which significant differences are found on only one range of the independent variable).

Exploratory analyses explored DRD4 x maternal sensitivity interactions predicting CBCL DSM subscales ADHD, affective, anxiety, and oppositional-defiant subscales, using similar adjusted linear regression models.

## Results

Table 1 shows demographics for our pilot cohort and associations of study variables by DRD4 genotype. *DRD4* genotype was unrelated to maternal age and depression, child sex, and maternal interactive sensitivity at 6-months postpartum in the overall sample (data not shown), and among European-ancestry children (Table 1). *DRD4* genotype was related to maternal sensitivity in the sub-sample of children with reported African ancestry (N = 21, Table 1). Mothers who reported African ancestry had lower levels of observed maternal sensitivity (on 5 point scale, mean maternal sensitivity was 3.7(0.68) in European-American vs 3.0(0.46) in African-American mothers, t(62) = 4,3. p < .001), but scores on CBCL were not different (not shown). Although the analysis of maternal *DRD4* genotype was not a primary focus of these analyses, these data were available. No significant or trend-level relationships were found between maternal *DRD4* genotype (7-allele carrier vs non-carrier) and maternal interactive sensitivity, maternal depression, nor child 18 months CBCL externalizing behaviors in the overall sample, nor among the European-ancestry and African-American ancestry subsamples analyzed separately (data not shown).

**Table 1:** Demographics of sample.

	European-only ancestry (N = 42)					African Ancestry (N = 21)				
		DRD4 7-allele					DRD4 7-allele			
	Non-carrier (N = 24)		7-allele carrier (N = 18)			Non-carrier (N = 10)		7-allele carrier (N = 11)		
	M	SD	M	SD	p	M	SD	M	SD	p
Maternal age	29.2	5.0	31.0	5.5	0.268	29.7	6.3	26.3	6.1	0.221
Maternal sensitivity (observed)	3.9	0.7	3.7	0.6	0.329	3.2	0.5	2.8	0.4	0.045
	N	%	N	%	p	N	%	N	%	p
Maternal depression (18 mo)	8	33.0%	3	17.0%	0.224	4	40.0%	7	63.0%	0.279
Male sex—child	9	38.0%	10	50.0%	0.245	5	50.0%	6	55.0%	0.835

**Fig 1:**
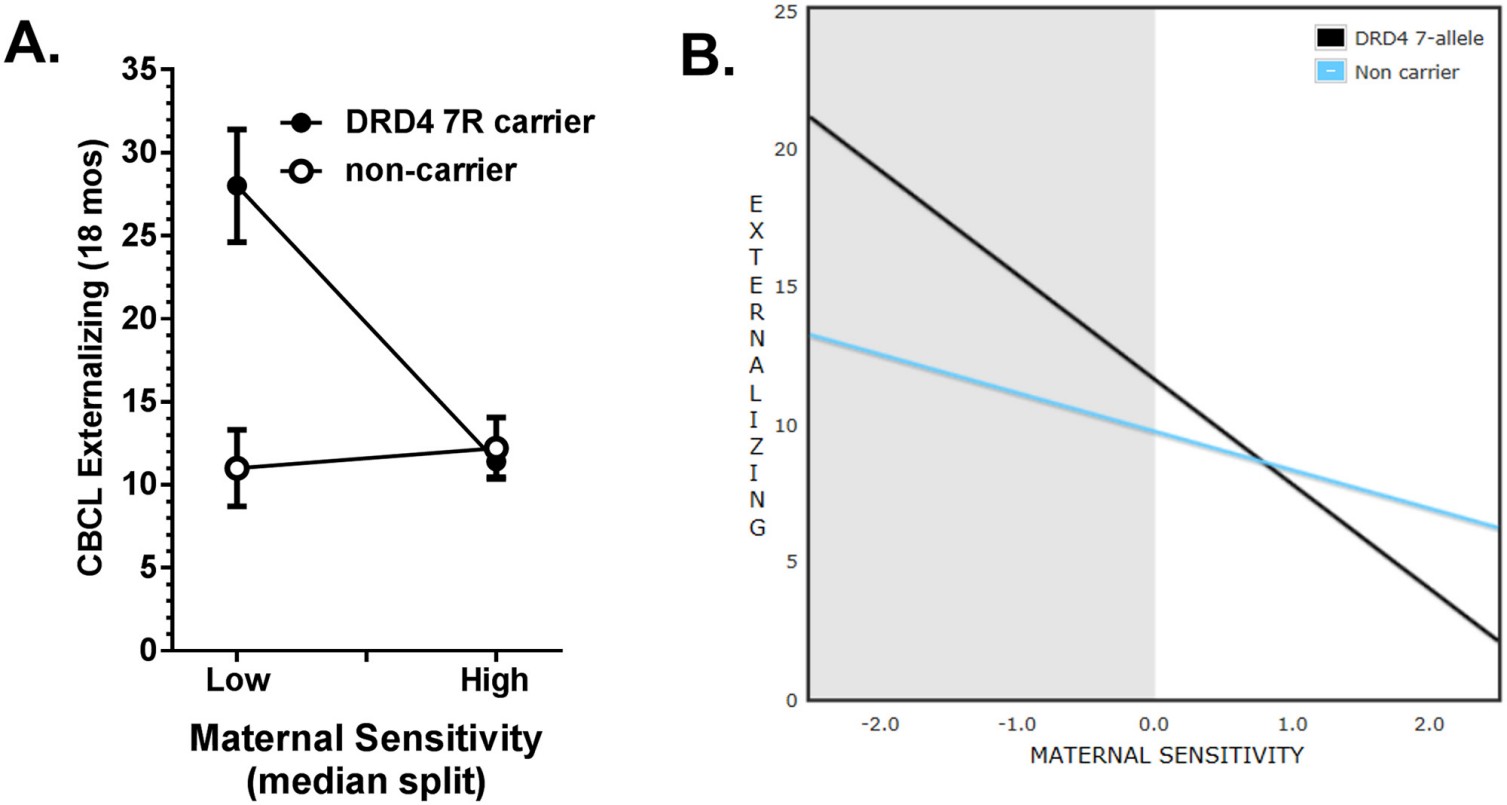
Interactive effect of *DRD4* genotype and maternal sensitivity at 6 months predicting externalizing behaviors at 18 months. **A.** CBCL Externalizing behaviors for European-ancestry toddlers at 18 months. *DRD4* 7R carriers or non-carriers, with mothers with high or low sensitivity (median split) at 6 months. **B.** Linear regression slopes of CBCL Externalizing behaviors at 18 months by Maternal Sensitivity at 6 months are depicted separately for 7R-allele carriers and non-carriers, controlled for maternal age, child sex, child ancestry, and maternal depression. Regions of significance (RoS) are highlighted in the graph (shaded area). Only the lower bound of Maternal Sensitivity falls within the RoS (i.e. significant *DRD4* differences in child externalizing behaviors are not seen in children with mothers expressing high sensitivity) consistent with a diathesis-stress model.

We first tested whether we could replicate the maternal sensitivity x *DRD4* 7R interaction effects on child Externalizing Behaviors child behavior problems previously reported by [22], using CBCL maternal report at 18 months. To parallel the prior study as much as possible, we first used the same analytic approach used in the previous report (i.e. median split of maternal sensitivity) in the portion of our sample of mother-infant dyads with only European ancestry (N = 42 dyads), and then in the entire sample (N = 63 dyads, combining dyads with European-only and African-ancestry children; separate analyses were not performed in the African-ancestry sample alone because of its size N = 21). In the European-only ancestry sample, ANOVA found significant main effects of maternal sensitivity median split (F(1, 38) = 13.8, p = .001, n2 = .266), *DRD4* genotype (F(1, 38) = 14.8, p < .001, n2 = .285), and a significant *DRD4* 7R x maternal sensitivity interaction predicting externalizing problems F(1,38) = 18.6, p = .00011, n2 = .329, (model F(3, 38) = 10.5, p = 3.63 x 10^−5^, adjusted *R*^*2*^ = .410). The *DRD4* x maternal sensitivity interaction remained significant after adding maternal depression at 18 months as a covariate (F(1, 37) = 25.0, p = 1.39 x 10^−5^, n2 = .404), model F(4, 37) = 10.8, p = 6.9 x 10^−6^, adjusted *R*^*2*^ = .488. Repeating the analysis in the entire sample of 63 dyads found main effects of maternal sensitivity (F(1, 62) = 4.8, p = .005, n2 = .128) and *DRD4* genotype (F(1, 62) = 5.9, p = .018, n2 = .092), and a significant *DRD4* 7R x maternal sensitivity interaction (F(1, 62) = 10.3, p = .002, n2 = .148, see Fig 1A). *DRD4* x maternal sensitivity interaction remained significant after adding maternal depression at 18 months as a covariate (F(1, 62) = 8.03, p = .001, n2 = .170) in the combined sample.

The *DRD4* x maternal sensitivity interaction for child Externalizing Problems was also significant in linear regression models controlling for potential confounds. Table 2 shows results from regression models of effects of maternal sensitivity (continuous measure), *DRD4* genotype, and *DRD4* 7R x maternal sensitivity on CBCL scales at 18 months, adjusted for maternal age, maternal depression at 18 months, child gender (and child African ancestry in the combined sample). Neither maternal sensitivity nor *DRD4* genotype had significant effects in an adjusted regression model without the interaction term. However, the *DRD4* x maternal sensitivity interaction term when added to the model (adjusted for main effects of genotype, maternal sensitivity, and potential confounds) had significant effects (betas) predicting 18 month CBCL externalizing problems in the European-only ancestry sample (N = 42, B = -3.42 SE = 1.33, p = .013), and in the total sample (N = 63, B = -2.15 SE = 0.92, p = .023). RoS analyses of the *DRD4* x maternal sensitivity interaction (see Fig 1B) found values of maternal sensitivity Z-score outside the bounds of -0.016 and 4.131 to be significant (i.e. the lower bound only of maternal sensitivity showed significant interaction). Tests of the difference in slopes at Z = -2.0 was t(62) = 3,23, p = .002; whereas the difference at Z = 2.0 was t(62) = 1.38, p = .173, non-significant. The Affected Proportion of the interaction was 0.77, indicating that 77% of the sample had values of maternal sensitivity in the range affected by the interaction.

**Table 2:** Replication Analysis: Linear regression of effect of DRD4 genotype, maternal sensitivity, and their interaction on 18 month child Externalizing Behavioral problems (Achenbach CBCL)^§^.

	*DRD4* genotype		Maternal Sensitivity		Interaction	
*sample*	beta	p	beta	p	Beta	p
European only (N = 42)^1^	0.265	0.105	-0.125	0.258	-.408*	0.013
All subjects (N = 63)^2^	0.228	0.053	-0.129	0.374	-.246*	0.023

^1^models controlling for maternal age, maternal depression at 18 mo, and child sex.

^2^models controlling for maternal age, maternal depression at 18 mo, child sex, and reported child African ancestry.

^§^*uncorrected p < .05

We then conducted exploratory analyses examining potential association of *DRD4* x maternal sensitivity interaction with DSM-oriented subscales of CBCL using similar adjusted linear regression models (Table 3). A *DRD4* x maternal sensitivity interaction effect was found on the ADHD subscale in the European-only sample (B = -0.94, SE = 0.38, p = .023) and in the total sample (B = -0.58, SE = 0.19, p = .002, which would survive Bonferonni correction for 8 multiple comparisons).

**Table 3:** Exploratory Analyses: Linear regression of effect of DRD4 genotype, maternal sensitivity, and their interaction on 18 month child DSM-problems subscales (Achenbach CBCL)^§^.

	*Achenbach CBCL*	*DRD4* genotype		Maternal Sensitivity		Interaction	
*sample*	*at 18 mo*	beta	P	beta	p	beta	p
	ADHD Problems	.283	.083	-.086	.599	-.349*	.023
European only	ODD Problems	.273	.101	-.080	.630	-.316	.069
(N = 42)^1^	Affective Problems	.201	.226	-.211	.211	-.328	.061
	Anxiety Problems	.059	.718	-.315	.067	-.312	.078
	ADHD Problems	.286*	.027	-.107	.458	-.317**	**.002**
All subjects	ODD Problems	.167	.183	-.035	.823	-.105	.403
(N = 63)^2^	Affective Problems	.169	.191	-.182	.260	-.099	.444
	Anxiety Problems	.155	.219.	-.237	.133	-.249*	.045

^1^models controlling for maternal age, maternal depression at 18 mo, and child sex.

^2^models controlling for maternal age, maternal depression at 18 mo, child sex, and reported child African ancestry.

^§^*uncorrected p < .05, **uncorrected p < .01.

Bolded: significant after Bonferoni correction.

## Discussion

We observed a significant interaction of child *DRD4* 7R allele carrier genotype and low maternal sensitivity (assessed by laboratory observation of mother-infant interactions at 6 months) that predicted child externalizing behavior problems at 18 months. Infants carrying at least one *DRD4* 7R allele with mothers showing insensitive parenting (lower 50% median split) had significantly higher CBCL externalizing behaviors (i.e., aggression, hyperactivity) and ADHD problems at 18 months. In sum, infants with both the genetic (i.e., 7R allele carrier) and contextual risk (i.e., with mothers displaying lower levels of sensitivity) showed more externalizing problem behaviors (i.e., aggressive, hyperactive, inattentive) in toddlerhood. Our data replicate previously reported findings of *DRD4* x maternal insensitivity in toddlers in European cohorts [22] [23]. Our study was designed as a planned independent replication of a previous report by [22], and thus we used comparable measures of maternal sensitivity and similar initial analysis (testing interaction term of 2 x 2 ANOVA with median split of maternal sensitivity). Similar to the previous study, we found a significant *DRD4* 7R x maternal sensitivity interaction to CBCL externalizing problems. The *DRD4* 7R x maternal sensitivity interaction was also significant in regression analyses using continuous measures of maternal sensitivity and adjusting for maternal age, maternal depression, and child ancestry. The analysis of RoS in the linear regression analyses implied that significant differences in child externalizing behaviors were found at low but not high levels of maternal sensitivity. That is, in this analysis *DRD4* 7-allele carrier children with mothers with low levels of parenting sensitivity had significantly greater externalizing behavior than non-carriers, but 7-allele carriers with mother with high levels of parenting sensitivity did not have significantly lower externalizing behavior. This would appear to be consistent with a “diathesis-stress” rather than a “differential susceptibility” model; that is, we did not find evidence of a beneficial effect of the 7-allele at high levels of maternal sensitivity. However, it should be noted that the N in this small replication study were low, and thus future studies with larger samples should further explore this possibility.

Our findings are also consistent with other reports of similar *DRD4* 7R x insensitive caretaking interactions associated with greater behavior problems in kindergarten [19], greater inattention in middle childhood [20], and greater ADHD, conduct disorder, and psychopathy at age 15 [21]. Thus the present data provide further evidence that children who carry the *DRD4* 7R allele appear to have increased susceptibility to effects of maternal sensitivity on behavior. It has been proposed that the carriers of the *DRD4* 7R allele are more sensitive to effects of caregiving and other environmental influences [14,33,34]. At present there are multiple clear examples of such G x E influences on child outcomes; for example, child maltreatment history interaction with 5-HTTLPR [35], *CRHR1* [17], *FKBP5* [16], and *ADRB2* [18] showing greater vulnerability to depression or PTSD as adults. The present study provides further support for G x E interaction with a more subtle form of potential childhood adversity, maternal parenting insensitivity, which creates risk for early childhood psychopathology.

We found significant *DRD4* 7R x maternal sensitivity interaction when children of both European and African ancestries were analyzed together, however the small number of children in this study, and in particular African-ancestry children, limit our ability to make inferences, and the potential roles of race / ancestry as a moderator of effects of maternal sensitivity and/or effects of *DRD4* 7R are yet to be fully elucidated. However, our findings are consistent with two reports from a group studying only European ancestry children, that found *DRD4* 7R- carriers appeared more susceptible to effects of low maternal sensitivity on externalizing behaviors [22,23]. However, another group that studied *DRD4* 7R allele x maternal sensitivity in both European and African ancestry children found potential differences in effects between ancestries [24]. Further work is needed in larger samples of children of European and other ancestries.

Previous work supports the notion that more sensitive parenting is associated with reduced risk for externalizing behavior problems in toddlers. Specifically, this prior work suggests that when parents are more sensitive, children develop more effective self-regulatory capacity reducing their risk for externalizing behaviors even when these children may have temperamental challenge [36] or genetic susceptibility [25]. When mothers are more sensitive, they are more effective in co-regulating their children’s affect and behaviors, ultimately leading to more effective self-regulation in toddlers and young children [37], which subsequently reduces risk in these children to display externalizing problem behaviors.

While here we were specifically interested in attempting to replicate previous findings of child *DRD4* genotype (carriage of the 7-allele) as a potential interactive risk factor with maternal insensitivity (i.e. child environment), we also recognize and are very interested in the possibility that maternal *DRD4* genotype could have effects on maternal attention, and thus also influence maternal interactive sensitivity. Since maternal and child *DRD4* genotype are necessarily related, this could contribute to one form of gene-environment correlation, such that child *DRD4* genotype is associated with maternal sensitivity. While the current replication sample is not sufficiently powered to definitively rule out such potential relationships, we did not detect significant (or trend-level) relationships between maternal sensitivity and maternal *DRD4* genotype in the total sample or either sub-sample. We also did not detect significant (or trend-level) relationships between maternal sensitivity and child *DRD4* genotype in the total sample or the European-ancestry sub-sample; a marginally significant difference in maternal sensitivity between the 7-allele carriers and non-carriers was seen in the African-American sub-sample only (N = 21), and thus future studies with larger sample sizes should further examine this trend. However, this trend cannot explain the *DRD4* x maternal sensitivity interaction seen in the European-ancestry children or the entire sample.

This small replication study has a number of limitations. The sample of mother-infant dyads was small (albeit of a similar sample size to original report), thus limiting power and possibly introducing some bias to the analyses. However, even with its small size, we detected similar significant interaction of the same direction as the initial study of (Bakermans-Kranenburg & van Ijzendoorn, 2006), and with a large effect size. Independent replication in unrelated samples is crucially important for validating and understanding the clinical meaning of genetic findings, and thus our study adds impact to previous findings. Bootstrapping was utilized in the regression analyses to also help offset problems of the small sample size. Another limitation may be that this current report is part of a longitudinal study in mothers oversampled for history of trauma and post-partum depression, and that some of the mothers in the sample had symptoms or diagnosis of depression at 6 months post-partum. Although maternal sensitivity was an observational objective assessment, child externalizing behaviors represented maternal ratings at 18 months, and could be biased by mother’s depression. Thus, all models were controlled for the presence maternal depression at the time of the maternal CBCL report (18 months), which did not appear to diminish the *DRD4* x maternal sensitivity interaction. Future studies would benefit from larger samples, laboratory coded measures of observed child behaviors, as well as behavioral reports from additional informants, such as teachers.

The direct replication of *DRD4* x maternal sensitivity interaction effect on Externalizing behaviors, using the same analytic approach in European-ancestry children only produced both a large effect size in the same direction as the original report [22], and a p-value of 1.1 x 10^−4^. While this p-value would survive Bonferoni correction for the total number of tests performed in this study (and is smaller than the original study), it was still not close to “genome-wide” significance (commonly accepted as ~5 x 10^−7^ in massive-parallel GWAS studies). However, replication of gene association findings is crucial for validation and interpretation [38], and as the present study was a planned replication using similar measures and analytic design, we argue, following prior examples [39] that even with the small N, we provide independent evidence for replication of this effect. We also provide evidence in exploratory analyses for a potential effect of *DRD4* x maternal sensitivity on the CBCL ADHD subscale, which would also remain significant after Bonferoni correction.

Finally, we are aware that this replication study examined only a single genetic variant, the *DRD4* VNTR, and that other genes may be influential as well. However, we chose to limit our focus on the *DRD4* gene for several reasons. First, the small sample would preclude concurrent analyses of multiple gene variants; also there is good evidence that the *DRD4* gene may be a particularly sensitive marker of environmental and/or social sensitivity [33,34,40,41]. Interestingly, haplotype analyses of the *DRD4* VNTR locus suggests that the 7R allele of *DRD4* may have arisen as recently as 40,000–50,000 years ago [41], and some have suggested it may be a site of selection [41,42],.

In summary, the current findings provide independent evidence to replicate findings of a *DRD4* 7R x maternal sensitivity interaction affecting externalizing behaviors in European-ancestry children, with 7R allele carriers having greater externalizing problems in the face of maternal insensitivity. This provides further evidence that gene variants such as *DRD4* VNTR may help to shape differential responses to environment influences in development, including such relatively subtle factors as the style of maternal caretaking behaviors. More work in larger samples utilizing objectively measured outcomes is needed to further validate these findings and expand them with more genetic information and in longitudinal cohorts, as in [23].
